# Prognostic value of right ventricular dilatation on computed tomography pulmonary angiogram for predicting adverse clinical events in severe COVID-19 pneumonia

**DOI:** 10.3389/fmed.2023.1213775

**Published:** 2023-07-31

**Authors:** Christophe Beyls, Jeremie Vial, Thomas Lefebvre, Charlotte Muller, Thomas Hanquiez, Patricia Besserve, Mathieu Guilbart, Guillaume Haye, Michael Bernasinski, Pierre Huette, Hervé Dupont, Osama Abou-Arab, Vincent Jounieaux, Yazine Mahjoub

**Affiliations:** ^1^Department of Anesthesiology and Critical Care Medicine, Amiens University Hospital, Amiens, France; ^2^Department of Radiology, Amiens University Hospital, Amiens, France; ^3^Department of Pneumology, Amiens University Hospital, Amiens, France

**Keywords:** right ventricle dilatation, AVDS, computed tomography, angiogram, COVID-19, pneumonia, ARDS

## Abstract

**Background:**

Right ventricle dilatation (RVD) is a common complication of non-intubated COVID-19 pneumonia caused by pro-thrombotic pneumonitis, intra-pulmonary shunting, and pulmonary vascular dysfunction. In several pulmonary diseases, RVD is routinely measured on computed tomography pulmonary angiogram (CTPA) by the right ventricle-to-left ventricle (LV) diameter ratio > 1 for predicting adverse events.

**Objective:**

The aim of the study was to evaluate the association between RVD and the occurrence of adverse events in a cohort of critically ill non-intubated COVID-19 patients.

**Methods:**

Between February 2020 and February 2022, non-intubated patients admitted to the Amiens University Hospital intensive care unit for COVID-19 pneumonia with CTPA performed within 48 h of admission were included. RVD was defined by an RV/LV diameter ratio greater than one measured on CTPA. The primary outcome was the occurrence of an adverse event (renal replacement therapy, extracorporeal membrane oxygenation, 30-day mortality after ICU admission).

**Results:**

Among 181 patients, 62% (*n* = 112/181) presented RVD. The RV/LV ratio was 1.10 [1.05–1.18] in the RVD group and 0.88 [0.84–0.96] in the non-RVD group (*p* = 0.001). Adverse clinical events were 30% and identical in the two groups (*p* = 0.73). In Receiving operative curves (ROC) analysis, the RV/LV ratio measurement failed to identify patients with adverse events. On multivariable Cox analysis, RVD was not associated with adverse events to the contrary to chest tomography severity score > 10 (hazards ratio = 1.70, 95% CI [1.03–2.94]; *p* = 0.04) and cardiovascular component (> 2) of the SOFA score (HR = 2.93, 95% CI [1.44–5.95], *p* = 0.003).

**Conclusion:**

Right ventricle (RV) dilatation assessed by RV/LV ratio was a common CTPA finding in non-intubated critical patients with COVID-19 pneumonia and was not associated with the occurrence of clinical adverse events.

## Introduction

Right ventricle (RV) dilatation (RVD) is a well-known complication of COVID-19 (1) and is associated with increased mortality (2). Understanding the pathophysiological mechanisms of the specific heart/lung interactions during COVID-19 infection is still in progress. Clinical, radiological, and histological data have demonstrated that COVID-19 pneumonia is an authentic pulmonary vascular disease due to pulmonary vascular endothelitis, capillary microvascular thrombosis, and neo-angiogenesis leading to intrapulmonary shunting, which define the so-called “Acute Vascular Distress Syndrome” (AVDS) (3). AVDS combines a dysregulation of pulmonary vascular tone promoted by pulmonary vascular endothelial dysfunction, pulmonary angiogenesis, and intrapulmonary shunting but also by pulmonary vasoconstriction due to hypoxemia and intravascular clot formation (3, 4). The consequence on the RV of these different pathophysiological mechanisms is an increase in RV loading conditions resulting in RVD often disproportionate to the severity of parenchymal lung damage (5).

Current guidelines recommend unenhanced chest computed tomography (CT) as the first line of investigation to assess the extent of COVID-19 pneumonia (6). Regarding the high cumulative incidence of acute pulmonary embolism (PE) in such cases, CT pulmonary angiogram (CTPA) was often proposed as the first line of COVID-19 pneumonia investigation (7). The right ventricle-to-left ventricle diameter ratio (RV/LV) is a simple, fast, reproducible CTPA parameter to assess RVD for risk stratification in patients with acute PE (8). An RV/LV ratio greater than 1 is known to predict clinical adverse events in acute pulmonary embolism, interstitial lung disease, and acute respiratory distress syndrome (9–11).

We hypothesize that the RVD on CTPA, outside the clinical situation of acute PE and mechanical ventilation, may represent an adaptation of the RV to the acute load changes related to the specific vascular involvement observed in AVDS. The aim of the study is to investigate the association between RVD, defined by an RV/LV ratio > 1 measured on CTPA, and adverse clinical events in a cohort of non-intubated COVID-19 patients admitted to ICU.

## Materials and methods

### Population

Adult patients (> 18 years old) admitted to our ICU for COVID-19 pneumonia with a CTPA performed within 48 h of ICU admission were included in the study. Patients were included on the day of CTPA performance. Exclusion criteria were pulmonary embolism confirmed on CTPA, patients under mechanical ventilation, or extracorporeal membrane oxygenation (ECMO) during the CPTA exam.

### Outcome

The primary outcome was the first occurrence of an adverse clinical event defined as ECMO, renal replacement therapy, or 30-day mortality after ICU admission.

### Data

Data from electronic files and medical reports were prospectively collected. SARS-CoV-2 infection was confirmed by RT-PCR on a nasopharyngeal swab or bronchoalveolar lavage at ICU admission. The simplified acute physiology score II (SAPS II) was used to evaluate the severity of illness at admission (12). Vasopressor use was assessed by the sequential organ failure assessment (SOFA) and cardiovascular SOFA score (SOFA cv) (13). The severity of acute respiratory distress syndrome (ARDS) was defined according to the Berlin definition (14). The different epidemic waves were characterized by three periods: (1) period A (between 28th February 2020 and 1st June 2020), (2) period B (between 1st September 2020 and 15th April 2020), (3) and the period C (between 12th December 2020 and February 2022).

### CTPA acquisition

**FIGURE 1:**
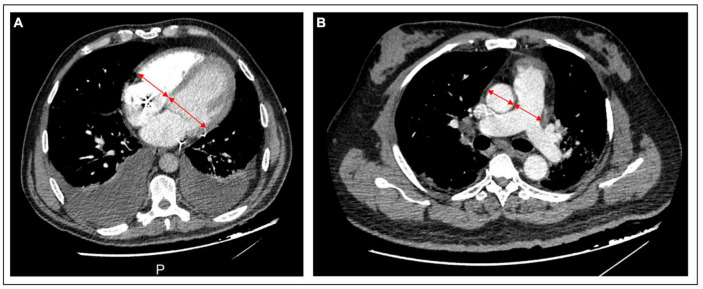
CTPA images of cardiac chambers and vessels measurement. **(A)** RV and LV basal diameters are measured by the maximum distance in diastole between the ventricular endocardium and the interventricular septum in a 4-chamber axial view. **(B)** In the axial view, the pulmonary artery and aorta diameter are measured by the broadest transverse diameter (inner wall-to-inner wall). CTPA, computed tomography pulmonary angiogram; LV, left ventricle; RV, right ventricle.

Computed tomography pulmonary angiogram (CTPA) was performed within 48 h of ICU admission using a 64-slice multidetector CT machine (General Electric Optima 660 or General Electric Discovery HD). All patients were non-intubated and examined in a supine position. A breath-hold was requested from the patients trying to avoid respiratory motion artifact. Bolus IV injection of non-ionic contrast medium was using an injector pump followed by 40 ml of saline solution. Rotation time was 0.33 s, 0.62 mm thickness, 0.7 reconstruction increment (mm), and 1 pitch. The images were transferred to the workstation (General Electric^®^, Milwaukee, WI, USA, AW 4.7), where the axial slides and multi-planar reformation were reviewed retrospectively by an experienced cardiothoracic radiologist (JV) blinded to clinical data. The following items were reported specifically for the study: (1) chest CT-SS for COVID-19 infection, (2) RV and LV diameters, (3) aorta diameter, and (4) pulmonary artery (PA) diameter (Figure 1).

### Chambers and vessels quantification on CTPA slides

The RV and LV basal diameters (maximum distance between the ventricular endocardium and the interventricular septum) were measured in a 4-chamber, axial and short-axis view. RV dilatation was defined as an RV/LV ratio > 1. At the level of the PA bifurcation, the inner wall-to-inner wall diameters of the aorta (AO) and the PA were obtained in the axial plane (PA measured just proximal to the bifurcation). PA dilatation was defined as a PA diameter > 29 mm (15). AO and PA diameter was measured on the same CTPA at its maximal diameter.

### CT-SS score

The severity of parenchymal involvement was assessed by the chest CT-SS as previously described (16). Segments of both lungs were divided into 5 regions and were visually scored on a scale of 1 to 5. Lung opacities were subjectively evaluated using system attributing scores: score 1 if the pulmonary involvement was less than 25%; score 2 if 26–50%; score 3 if 51–74%; score 4 if more than 75%. The highest total CT-SS score was 20.

### Echocardiographic measurements

Echocardiographic data of non-intubated patients (*n* = 76) with *trans*-thoracic echocardiography (TTE) performed within 48 h of ICU admission were analyzed. Standard echocardiography protocol was used according to the American Society of Echocardiography guidelines (17) and the European Society of Cardiology (18). Echocardiographic images were obtained through a high-quality and commercially available ultrasound system (CX 50, Philips Healthcare, Andover, MA, USA). RV systolic function was assessed by conventional parameters such as tricuspid annular plane systolic excursion (TAPSE), tricuspid S wave (RV-S’), and RV fractional area change (RV-FAC) in apical four chamber-view as recommended (17).

### Statistical analysis

Data are expressed as mean ± standard deviation (SD), median [interquartile range], or numbers (percentage), as appropriate. First, the overall population was divided into two groups according to the presence of RVD defined by an RV/LV ratio > 1. Variables were compared between groups (RVD and non-RVD group) using Mann–Whitney or Chi-square tests. We performed a Spearman correlation analysis to assess the association between the RV/LV ratio, measured on CTPA and TTE, and the PaO_2_.

Univariate and multivariate Cox models were performed to evaluate independent factors associated with adverse clinical events. All factors with a *p*-value < 0.05 in univariate analysis were included in the Cox model. Delong’s test compared the area under Receiving operative curves (ROC) (AUC) of RVD parameters on CTPA and chest CT-SS for identifying adverse events. The Kaplan–Meier method was used to plot the survival curves between the two groups and compared with the log-rank test.

To assess the intra-operator and inter-operator reproducibility of chamber quantification and vessel diameters, data from 10 patients were randomly selected and analyzed by the same operator and another operator with an interval of at least 1 week between the two analyses. The reproducibility of CTPA parameters was evaluated using the intraclass correlation coefficient (ICC). A statistical test was significant when the *P*-value was under 0.05. All *P*-values are the results of 2-tailed tests. Statistical analyses were performed using SPSS software version 24 (IBM Corp., Armonk, NY, USA).

## Results

Between February 2020 and February 2022, 403 non-intubated patients were admitted to Amiens University Hospital ICU for COVID-19 pneumonia. Among them, 157 patients (39%) were discarded due to a CTPA not being performed within 48 h of ICU admission, 57 (14%) for mechanical ventilation before CTPA and 8 (0, 4%) were excluded for PE on CTPA. Thus, a total of 181 patients were included in the study. Patients were divided into two groups according to the presence or the absence of an RVD defined by an RV/LV ratio > 1. We found a RVD in 112 patients (62%), whereas no RVD was noted in the remaining 69 patients (38%) (see Supplementary Figure 1).

### Clinical, demographic characteristics of patients and CTPA parameters

There were no significant differences between the two groups (RVD and non-RVD) regarding age, SAPS II score, clinical presentation, and biology at inclusion (Table 1).

**TABLE 1:** Characteristics and clinical outcomes of the study population.

Variables	No RVD (*n* = 69)	RVD (*n* = 112)	*P*-value
Age, (years)	59 [59–67]	59 [57–69]	0.83
BMI, (kg.m^–2^)	30.1 [25.9–33.8]	30.1 [26.1–34.2]	0.95
SAPS II	30 [23–41]	29 [22–47]	0.92
Male, *n* (%)	43 (62)	70 (62)	1
**Medical history *n* (%)**			
None	8 (11)	13 (12)	1
Hypertension	32 (46)	58 (52)	0.54
Diabetes	23 (33)	30 (27)	0.41
Dyslipidemia	20 (29)	32 (29)	1
Smoker	11 (16)	19 (17)	1
Chronic kidney disease	7 (10)	11 (10)	1
COPD	7 (10)	8 (7)	0.58
Asthma	4 (6)	7 (6)	1
Obstructive sleep apnea	4 (6)	11 (10)	0.41
Chronic coronary syndrome	7 (10)	11 (10)	1
Peripheral arterial disease	8 (12)	10 (9)	0.61
**Epidemic wave *n* (%)**			
Period 1	11 (16)	18 (16)	1
Period 2	17 (25)	27 (24)	1
Period 3	41 (59)	67 (60)	1
Time from symptom onset to admission to ICU, (day)	8 [5.5–12]	8 [5–10]	0.49
**Biological data at the inclusion day**			
pH	7.44 [7.38–7.47]	7.42 [7.34–7.46]	0.28
Pa0_2_, (mmHg)	72 [61–87]	73 [60–91]	0.73
P/F ratio	94 [79–164]	116 [81–153]	0.51
PCO_2_, (mmHg)	34 [31–40]	37 [32–41]	0.12
Lactate, (mmol.l^–1^)	1.8 [1.3–2.1]	1.6 [1.1–2.0]	0.11
Lymphocyte count (x10^6^ l^–1^)	600 [475–900]	700 [500–900]	0.26
Serum creatinine, (μmol.l^–1^)	64 [50–88]	69 [54–88]	0.34
C reactive protein, (mg.l^–1^)	131 [72–206]	127 [86–181]	0.88
Platelet count, (x10^9^ l^–1^)	221 [192–284]	228 [151–304]	0.43
Fibrinogène, (g.l^–1^)	5.6 [4.7–6.8]	5.9 [4.8–7.1]	0.42
PT (%)	80 [68–89]	76 [70–87]	0.41
aPTT	1.05 [0.95–1.19]	1.08 [0.97–1.22]	0.64
Troponin Tc HS, (ng.ml^–1^)	17 [8–44]	17 [6–47]	0.52
BNP, (pg.ml^–1^)	66 [23–122]	73 [28–206]	0.52
**Clinical course in ICU**			
Endotracheal intubation	32 (46)	48 (43)	0.64
Time from MV to ICU admission, (days)	3 [1–6]	2 [1–4]	0.24
Severe ARDS	28 (41)	35 (32)	0.20
Duration of MV, (days)	22 [10–31]	18 [10–28]	0.63
Ventilator-associated pneumonia	31 (97)	46 (41)	0.64
Pneumothorax	8 (12)	6 (5)	0.15
Occurrence of acute cor pulmonale	7 (10)	22 (20)	0.18
SOFA cv > 2*	22 (32)	27 (24)	0.49
Pulmonary embolism	6 (9)	8 (7)	0.77
**Adverse clinical events, *n* (%)**	22 (32)	32 (29)	0.73
Renal replacement therapy	12 (17)	12 (10)	0.19
ECMO	6 (9)	9 (8)	1
30 days mortality	17 (24)	18 (16)	0.17

Data are presented as median [interquartile range] and number (percentage). *SOFA cv > 2 was defined by the administration of norepinephrine or epinephrine. RVD, right ventricle dilatation; aPTT, activated partial thromboplastin time; ARDS, acute respiratory distress syndrome; BMI, body mass index; BNP, brain natriuretic peptide; CT, computerized tomography; COPD, chronic obstructive pulmonary disease; CV, cardiovascular; ECMO, extracorporeal membrane oxygenation; MV, mechanical ventilation; PT, prothrombin time; SAPS, simplified acute physiology score; SOFA, sepsis-related organ failure assessment; WBC, white blood cell.

**FIGURE 2:**
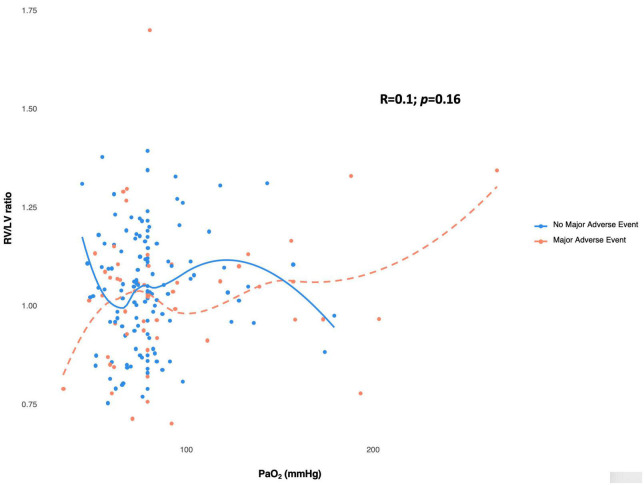
Scatterplot showing the heterogeneous relationship between Pa0_2_ and RV/LV ratio measured in CTPA. RV/LV ratio and PaO_2_ do not demonstrate a monotonic correlation (Spearman’s correlation coefficient = 0.1). The absence of a correlation exists independent of no occurrence of major adverse event (blue circles) or occurrence of major adverse event (red circle). The spline curves plotting RV/LV ration against PaO_2_ for patients with no major adverse event are shown by the blue line, while the patients with major adverse event are represented by the dashed red line. These curves demonstrate no significant change in slope across the range of observed values. LV, left ventricle; RV, right ventricle.

Computed tomography pulmonary angiogram (CTPA) parameters are presented in Table 2. For the overall study population, the mean RV/LV ratio was 0.91 ± 0.2. The relationship between RV/LV ratio and PaO_2_ was heterogenous (Figure 2), and the two variables were not correlated (Spearman’s correlation coefficient = 0.1, *P* = 0.16).

**TABLE 2:** CTPA and TTE features according to the presence of RVD.

Variables	No-RVD (*n* = 69)	RVD (*n* = 112)	*P*-value
Alveolar condensation, *n* (%)	38 (55)	61 (54)	1
Crazy paving, *n* (%)	19 (27)	27 (24)	0.86
Chest CT-SS	10 [5–15]	10 [10–15]	0.31
Left ventricle diameter, (mm)	47 [43–52]	43 [40–47]	0.001
Right ventricle diameter, (mm)	41 [37–46]	48 [44–52]	0.001
RV:LV ratio	0.88 [0.84–0.96]	1.10 [1.05–1.18]	0.001
Main PA diameter, (mm)	28.1 [26–30.7]	29.3 [26.5–33.3]	0.12
Dilatation of the PA, *n* (%)	27 (39)	62 (55)	0.045
Aorta diameter, (mm)	33.7 [29.2–36.4]	33.6 [30.7–36.5]	0.65
PA:AO ratio > 1	13 (19)	16 (14)	0.53
**TTE RV Parameters (*n* = 76)**			
RV basal dimension (mm)	43 [39–52]	44 [41–51]	0.73
RV mid-cavity dimension (mm)	35 [27–41]	36 [32–41]	0.87
RV longitudinal dimension (mm)	55 [70–88]	78 [70–85]	0.61
RV EDA (cm^2^)	18.7 [15.2–26]	21.3 [18.7–26.0]	0.17
RV ESA (cm^2^)	10.5 [7.3–14.2]	12.8 [9.2–16.4]	0.09
RV/LV ratio	0.84 [0.56–1.01]	0.95 [0.71–1.32]	0.04
RA volume (ml)	32 [18–50]	46 [27–57]	0.03
**TTE RV systolic function parameters**			
TAPSE (mm)	22 [20–25]	24 [18–27]	0.45
RV- S’ wave (cm/s-1)	16 [14–20]	17 [13–19]	0.93
RV-FAC (%)	45 [40–53]	45 [38–49]	0.62

Data are presented as median [interquartile range] and number (percentage). AO, aorta; CT-SS, computerized tomography severity score; FAC, fractional area change; LV, left ventricle; TAPSE, tricuspid annular plane systolic excursion; PA, pulmonary artery; RV, right ventricle; RVD, right ventricle dilatation.

In the RVD group, the median ratio of RV/LV was 1.10 [1.05–1.18], with more patients with PA dilatation than in the non-RVD group (*n* = 62, 55%, vs. *n* = 27, 39%; *P* = 0.045). No differences were found between the two groups for AO diameter, PA/AO ratio, and CT-SS score (all *p* > 0.05). Adverse clinical events occurred in 54 patients: 32 (28%) in the RVD group and 22 (32%) in the non-RVD group (*p* = 0.73). There was no difference in the occurrence of renal replacement therapy (RRT), ECMO, and 30-day ICU mortality between the two groups (all *p* > 0.05).

### Echocardiographic findings

Among the 181 patients, 76 (42%) had TTE within 48 h of ICU admission: 28 patients (41%) were in the non-RVD group, and 48 patients (43%) were in the RVD group (see Supplementary Table 1). The two groups appeared similar regarding left ventricular systolic and diastolic function. In the RVD group, the RV/LV ratio (0.95 [0.71–1.32] vs. 0.84 [0.56–1.01]; *P* = 0.04) and the right atrial (RA) volume (46 [27–57] ml vs. 32 [18–50] ml; *P* = 0.03) are higher in the RVD group. RV systolic function assessed by conventional parameters was similar in the two groups. The relationship between RV/LV ratio measured on TTE and PaO_2_ was heterogeneous (Supplementary Figure 2), and the two variables were not correlated. In a subgroup analysis, we dichotomized the population according to the presence of RVD as defined on TTE by a RV/LV ratio > 1 (Supplementary Table 2). We did not observe any significant differences in demographic characteristics, biological data, clinical course, and occurrence of adverse clinical events.

### Cox analysis

**FIGURE 3:**
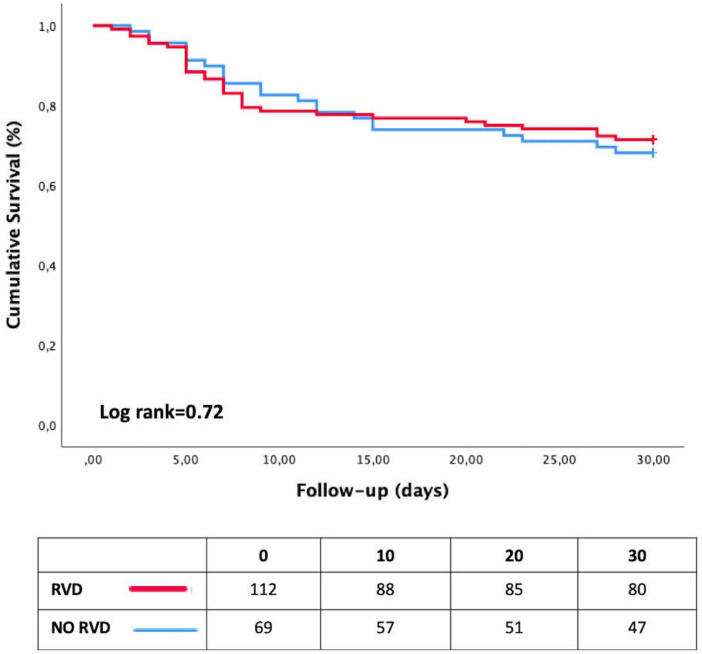
Kaplan–Meier curves showing event-free survival according to the presence of right ventricular dilatation. RVD, right ventricle dilatation.

On Cox univariate analysis, age > 65 years (HR = 2.48; 95% CI [1.45–4.23]; *p* = 0.001); hypertension (HR = 1.97; 95% CI [1.13–3.45]; *P* = 0.02); chronic renal failure (HR = 2.24; 95% CI [1.31–4.94]; *p* = 0.006); severe ARDS {HR = 3.48; 95% CI [2.01–6.01]; *p* = 0.0001; SOFAcv > 2 (HR = 4.74, 95% CI [2.75–8.16]; *p* = 0.001)} and chest CT-SS > 10 (HR = 1.78; 95% CI [1.04–3.01]; *p* = 0.03) were associated with the occurrence of adverse clinical events. After multivariable adjustment, the SOFA cv and the chest CT-SS > 10 remained independently associated with the occurrence of an adverse event (all *p* < 0.05) (Table 3). The analysis of survival curves showed no differences between the two groups (log Rank *P* = 0.72; Figure 3).

**TABLE 3:** Univariate and multivariate Cox regression analysis of predictive variables associated with adverse clinical events in patients with COVID-19 pneumonia.

Variables	Adverse clinical events			
	**Univariate analysis**		**Multivariate analysis**	
	**HR (95% CI)**	***P***	**HR (95% CI)**	***P***
Age > 65 years	2.48 [1.45–4.23]	0.001	1.58 [0.89–2.81]	0.11
BMI > 30	0.77 [0.41–1.35]	0.74	–	–
Hypertension	1.97 [1.13–3.45]	0.02	1.36 [0.74–2.52]	0.31
COPD	0.64 [0.21–2.01]	0.46		
Diabetes	1.61 [0.97–2.27]	0.09		
Chronic kidney failure	2.45 [1.31–4.94]	0.006	1.61 [0.78–3.28]	0.19
Severe ARDS	3.48 [2.01–6.01]	0.0001	1.61 [0.78–3.32]	0.19
Chest CT-SS > 10	1.78 [1.04–3.01]	0.03	1.71 [1.03–2.94]	0.04
PA/Aorta ratio > 1	0.51 [0.29–1.26]	0.14	–	–
RV/LV ratio > 1	0.91 [0.52–1.56]	0.72	–	–
PA > 29 mm	0.85 [0.51–1.46]	0.56	–	–
SOFA cv > 2	4.74 [2.75–8.16]	0.001	2.93 [1.44–5.95]	0.003
Use of dexamethasone	1.35 [0.67–2.69]	0.39	–	–

ARDS, acute respiratory distress syndrome; BMI, body mass index; COPD, chronic obstructive pulmonary disease; CT-SS, computed tomography severity score; CV, cardiovascular; HR, hazard ratio; LV, left ventricle; PA, pulmonary artery; RV, right ventricle; SOFA, sepsis-related organ failure assessment.

### CTPA parameters

No predictive factors of RVD were found in our study. The comparison of ROC curve analysis showed no differences between the different CTPA parameters to predict adverse clinical events (Supplementary Figure 3). The reproducibility of RV/LV ratio, AO, and AP diameter measurements was excellent, with an ICC > 0.8 for the intra and inter-operator reproducibility (see Supplementary Table 3).

## Discussion

Our study evaluating the RVD on CTPA in a cohort of non-intubated COVID-19 patients hospitalized in ICU found the following results: (1) the incidence of RVD on CTPA was 62%; (2) RVD was not associated with the occurrence of adverse clinical events, to the contrary to a SOFAcv > 2 and a chest CT-SS > 10; (3) RVD was not related to the severity of lung parenchymal involvement or myocardial injury and (4) CTPA parameters were highly reproducible.

### RVD on CTPA

Right ventricle dilatation (RVD) is a common feature of COVID-19 pneumoniae (19); whether it is evaluated on CT or echocardiography, RVD is frequently present in the initial phase of the disease (20). In our study, 62% (*n* = 112/181) of patients with COVID-19 pneumonia had RVD defined by an RV/LV ratio > 1 with a mean of 1.10 [1.05–1.18]. Our results are close to those of Rossdale et al. (5) who reported in a study a RVD (diagnosed on CTPA) prevalence of 59% in hospitalized COVID-19 patients on CTPA (*n* = 206/351) with a mean RV/LV ratio of 1.06 in the non-PE group. In the PE group (*n* = 56/351, 16%), the mean RV/LV ratio was at 1.17 (*p* = 0.011). In another study by Tao et al. (21) CTPA was performed in 100 non-intubated COVID-19 patients without PE. The authors found a RV/LV ratio of 1.06 ± 0.14 in patients who developed adverse clinical events (21). In our study, the mean RV/LV ratio was 0.91 [0.71–1.13], similar to that of the Suarez Castillejo et al. (22) study, which reported in a cohort of 179 patients with CTPA a RV/LV ratio of 0.9 [0.9–1.0] with no difference between the PE group (*n* = 76/179, 42%) and the non-PE group (*n* = 105/179, 58%).

### RVD and the occurrence of adverse clinical events

In our study, RVD defined by the RV/LV ratio > 1 was not associated with adverse clinical events contrary to the CT-SS score and the SOFA cv > 2. Even if the association between the RV/LV ratio and mortality has been firmly established in acute PE or interstitial lung disease (10, 23), data regarding COVID-19 pneumoniae remain discordant.

In the study of Rossdale et al. (5) RVD (defined by the RV/LV ratio > 1) was not associated with 30-day mortality. Besides, the RV/LV ratio at admission failed to identify patients who will die, and no optimal RV/LV ratio cut-off was established (5). In the Suarez Castillejo et al. (22) study, patients with COVID-19 and acute PE presented an elevated RV/LV ratio (mean at 1.0) without any association with ICU admission and mortality. In Tao et al. (21) study, patients with adverse clinical events had a higher RV/LV ratio (1.06 ± 0.14 vs. 0.95 ± 0.15, *p* < 0.001). Moreover, the authors found that a cut-off value of 1.1 is a potential marker of adverse outcomes. However, 45% (*n* = 24/53) of the adverse events were related to “ICU admission,” which makes results interpretation and comparison challenging (16).

Chest CT-SS and the cardiovascular component of the SOFA score are well-known parameters of early clinical severity in COVID-19 pneumonia. In an international multicentric study of 424 COVID-19 patients hospitalized in ICU, a SOFA cv ≥ 3 was independently associated with 90 days ICU mortality (HR = 1.79, 95% CI [1.52–2.11], *P* < 0.001). However, RVD and CT-SS were not reported in this study (24). The chest CT-SS was developed for assessing the COVID-19 burden on the initial chest CT obtained at hospital admission (25). Several definitions and calculation methods have been developed for the chest CT-SS score, but all were correlated with mortality in COVID-19 disease (26). As in recent studies, we found that RVD was not associated with chest CT-SS which evaluated the lung lesion burden of COVID-19 pneumoniae.

### No association between CT-SS, RV dysfunction, myocardial injury, and RVD

In COVID-19 disease, RV myocardial injury or lung injury (myocardial infarction, direct viral injury, hypoxia, ARDS, pulmonary thrombosis, and inflammatory injury) may lead to RV dysfunction (19). It has been previously shown that RV dysfunction in COVID-19, especially in the case of acute core pulmonale, was associated with a higher mortality risk (20). The absence of a relationship between RVD and the severity of parenchymal lung involvement suggests that RVD cannot only be attributed to an increased pulmonary vascular resistance (PVR) due to hypoxic pulmonary vasoconstriction. Moreover, RVD does not appear to be associated with myocardial ischemic injury or impairment of RV contractility. Indeed, RVD was not associated with biomarkers of myocardial injury or heart failure. Moreover, RV systolic function, measured by TTE, was preserved despite RVD.

One hypothesis is that, in the early phase of the disease, RVD corresponds to a physiological RV adaptation coping with the increase of pulmonary vascular volumes (27) due to pulmonary neoangiogenesis and bronchial and/or pulmonary vessels dilatation encountered in the COVID-19 pneumoniae (28). Several studies have emphasized that COVID-19 is characterized by acute lung neoangiogenesis and bronchial and pulmonary vascular dilatation. This has been shown in histopathological studies and in pulmonary imaging studies (28–30). Regarding hemodynamics, Caravita et al. (31) have shown that COVID-19 patients have a higher cardiac index and lower pulmonary vascular resistance (PVR) than patients with non-COVID-19 pneumonia of similar gravity. We suggested that the consequences of these acute changes are similar to those of the chronic changes observed during hepato-pulmonary syndrome (32, 33). Hence, in our study, RVD appears to be independent of PaO_2_ and from parenchymal lung involvement in COVID-19 patients without PE. Moreover, RVD does not have any prognostic value in this population. According to these findings, we suggest that RVD is an adaptive response to the vascular modifications related to COVID-19.

### Limits

First, we acknowledge that the main limitations of our study are the small sample size and its monocentric character. However, to the best of our knowledge, it is the first study to assess the clinical impact of the RVD defined by the RV/LV ratio measured by CTPA on critically ill COVID-19 patients at the early stage of the disease.

Second, we could not analyze the clinical impact of the different SARS-CoV-2 “variants of concern” (VOC). In our center, systematic screening for SARS-CoV-2 mutations was only performed after November 2020. The pathogenicity of the dominant VOC during the inclusion period likely impacts the clinical course of patients (18).

Third, the impact of patients with potentially pre-existent RVD was not evaluated. However, the incidence of COPD and asthma was very low in our cohort, thus reducing a potential selection bias.

Lastly, we excluded patients under mechanical ventilation and ECMO because these two organ support techniques modify the RV afterload, making interpretation of RVD in the initial phase of the disease challenging.

## Conclusion

Right ventricle dilatation (RVD), defined by the RV/LV ratio > 1, is a common finding on CTPA in non-intubated patients hospitalized in ICU for COVID-19 pneumonia. RVD, a probable adaptive phenomenon, does not appear to be associated with adverse clinical events and should not be used for risk stratification in the early phase of COVID-19 pneumonia without pulmonary embolism.

## Data availability statement

The raw data supporting the conclusions of this article will be made available by the authors, without undue reservation.

## Ethics statement

The studies involving human participants were reviewed and approved by the Amiens University Hospital Board. Written informed consent was not provided because French law on clinical research for non-interventional studies, informed consent was waived. Still, oral and written information was provided whenever possible to the patients and systematically to their families, specifying that they could oppose the use of their data (34).

## Author contributions

CB, OA-A, and YM: study conception and manuscript drafting. JV, CM, TH, PB, MB, MG, and PH: clinical data collection. CB and YM: statistical analysis. CB, YM, HD, and VJ: manuscript revision. All the authors approved the final version of the manuscript.
