# Supplementary material for: Prognostic value of right ventricular dilatation on computed tomography pulmonary angiogram for predicting adverse clinical events in severe COVID-19 pneumonia
Source: Front Med (Lausanne). 2023 Jul 31;10:1213775. doi: 10.3389/fmed.2023.1213775 (PMC10425267; doi:10.3389/fmed.2023.1213775)
Supplement: Supplementary file 1 [file Data_Sheet_1.docx]

**Supplementary Files**


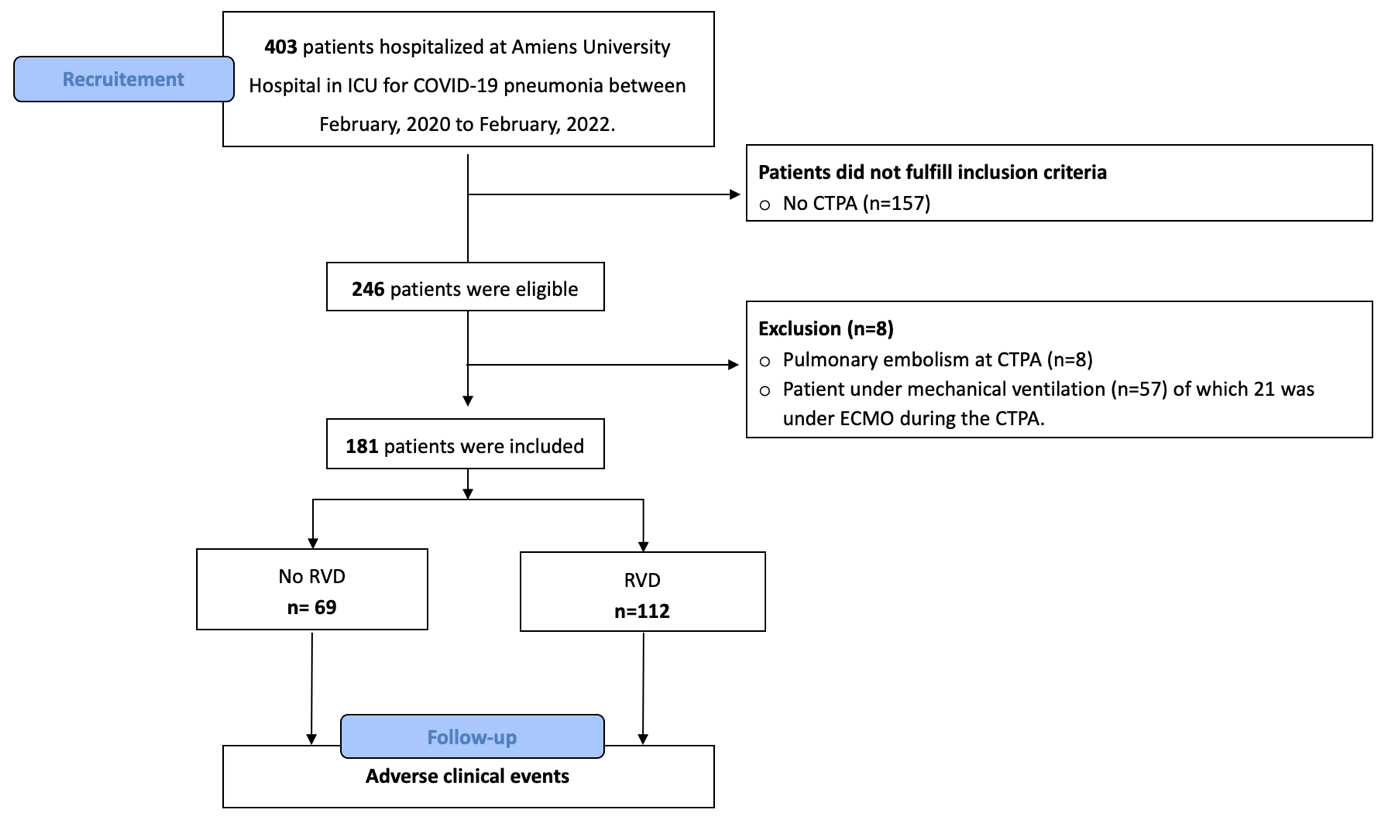


**Figure 1 Supplementary Files: Flow chart of the study.**

**CTPA**: computed tomography pulmonary angiogram; **ECMO**: extracorporeal membrane oxygenation; **ICU**: intensive care unit; **RVD**: right ventricle dilatation.

| **Overall population (n=76)** | **No RVD**  **(n=28)** | **RVD**  **(n=48)** | **P value** |
| --- | --- | --- | --- |
| ***LV systolic parameters***  LVEF (%)  LV end diastolic volume (ml)  LV end systolic volume (ml)  Stroke volume (ml.s^-1^)  CO (l.min^-1^) | 61 [56-70]  81 [56-118]  29 [20-48]  61.4 [48.8-76]  5.3 [4.1-6.8] | 61 [50-72]  106 [67-123]  38 [23-55]  63 [50.1-85.8]  4.5 [3.8-5.6] | 0.72  0.19  0.23  0.36  0.11 |
| ***LV diastolic functional parameters***  E wave (cm s^-1^)  A wave (cm s^-1^)  E/A ratio  Lateral E/e’  E wave deceleration time (ms) | 80 [70-100]  78 [62-101]  1.0 [0.8-1.5]  9.0 [7.6-12.1]  257 [197-310] | 81 [54-97]  70 [52-98]  0.87 [0.65-1.44]  8.2 [5.6-10.1]  271 [202-334] | 0.54  0.79  0.33  0.08  0.79 |
| LA volume (ml) | 35 [17-44] | 44.6 [30.1-53.1] | 0.15 |
| Major adverse events | 12 (36) | 19 (33) | 0.95 |

**Table 1 – Echocardiography data**

Continuous variables are expressed as median [interquartile range]

**CO:** cardiac output; **LA:** left atrial; **LV:** left ventricle. **LVEF:** left ventricular ejection fraction;


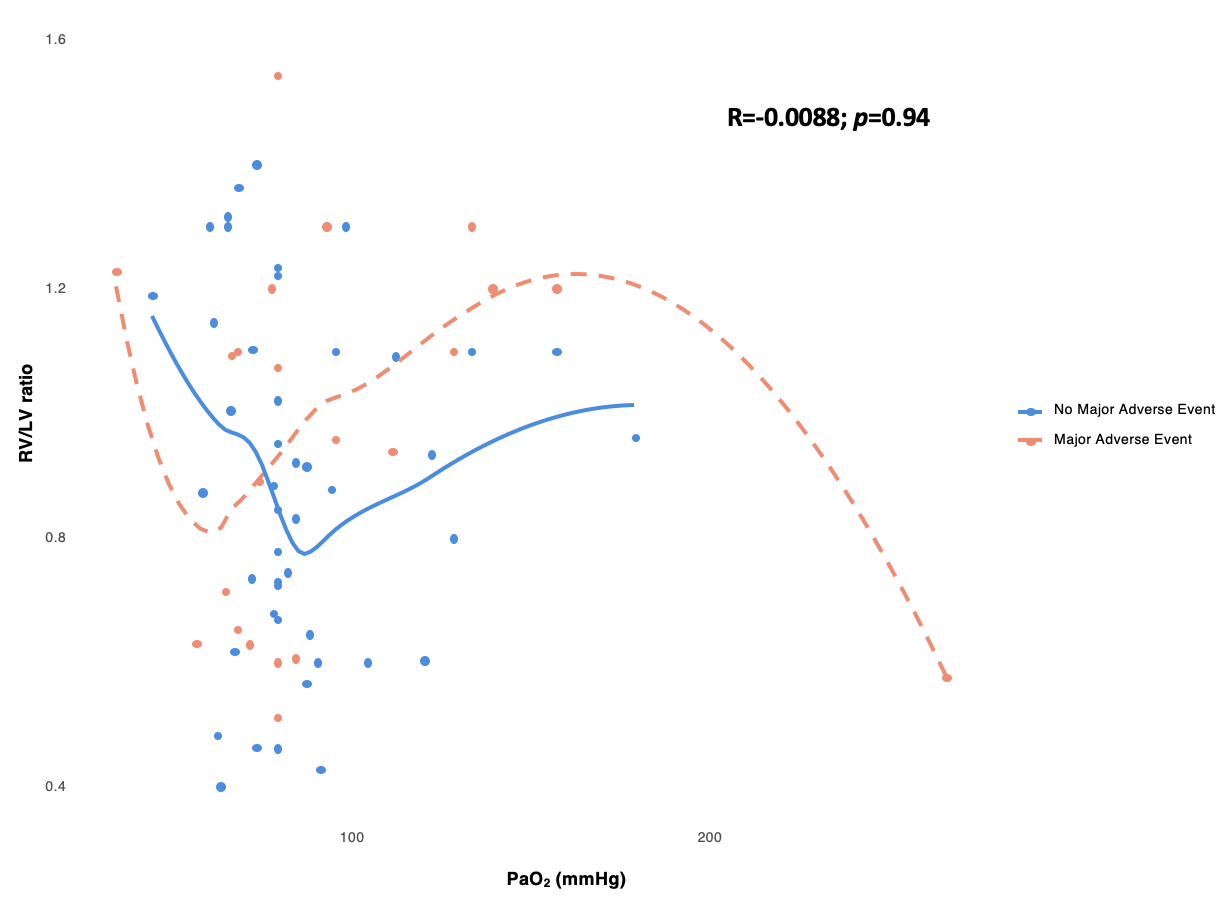


**Figure 2 – Scatterplot showing the heterogeneous relationship between Pa0_2_ and RV/LV ratio measured in TTE.**

**LV:** left ventricle; **RV:** right ventricle.

**Table 2 – Demographic and clinical characteristics according to the presence of RVD as defined on TTE**

| **Variables** | **No RVD**  **(n=40)** | **RVD**  **(n=28)** | **P value** |
| --- | --- | --- | --- |
| Age, (years) | 59 [59-68] | 59 [59-69] | 0.676 |
| BMI, (kg.m^-2^) | 30 [27-34] | 31 [28-36] | 0.119 |
| SAPS II | 28 [24-45] | 32 [26-48] | 0.499 |
| **Biological data at the inclusion day**  pH  Pa0_2_, (mmHg)  P/F ratio  PCO_2,_ (mmHg)  Lactate, (mmol.l^-1^)  Troponine Tc HS, (ng.ml^-1^)  BNP, (pg.ml^-1^) | 7.42 [7.30-7.46]  79 [73-91]  94 [78-139]  37 [33-45]  1.9 [1.1-2.3]  20 [8-42]  64 [38-106] | 7.41 [7.32-7.45]  78 [66-97]  116 [90-140]  39 [33-45]  1.8 [1.3-2.0]  11 [5-23]  72 [38-174] | 0.85  0.46  0.65  0.52  0.83  0.18  0.71 |
| **Clinical course in ICU, n (%)**  Endotracheal intubation  Ventilator-associated pneumonia  Pneumothorax  SOFA cv >2  Pulmonary Embolism  Adverse clinical events | 21 (46)  22 (48)  3 (7)  10 (22)  1 (2)  14 (30) | 14 (47)  12 (40)  4 (13)  9 (30)  0  12 (40) | 1  0.66  0.42  0.62  1  0.54 |

Data are presented as median [interquartile range] and number (percentage).

**RVD:** right ventricle dilatation**; BMI:** body mass index. **BNP:** brain natriuretic peptide; **SAPS:** simplified acute physiology score; **SOFA**: sepsis-related organ failure assessment.


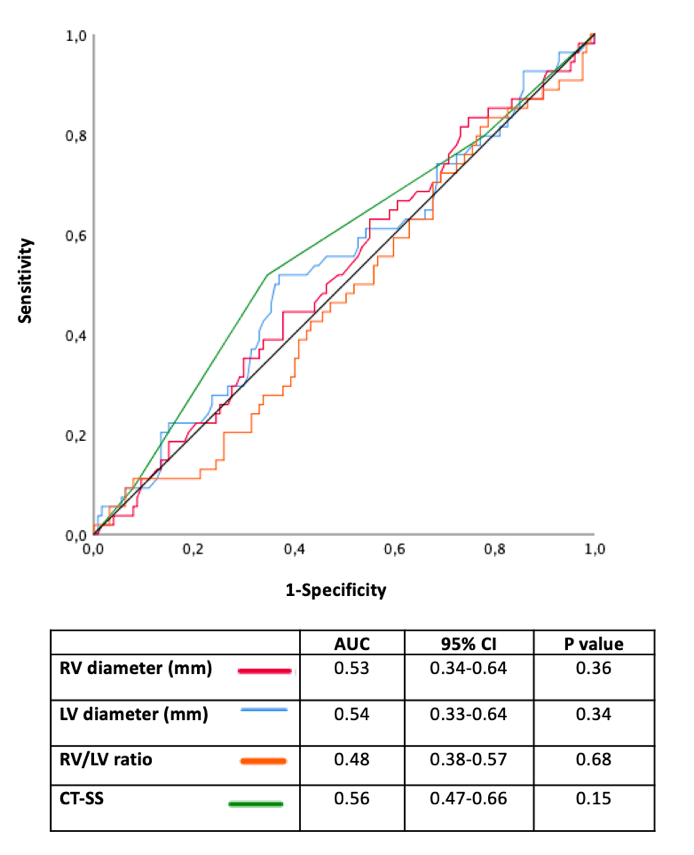


**Figure 3 – Receiving operative characteristic curve of CTPA parameters for identifying patients with adverse clinical events**

**AUC:** area under the curve; **CI**: confidence interval; **CT-SS:** computed tomography severity score; **LV:** left ventricle; **RV:** right ventricle.

**Table 3 – Reproducibility of CTPA parameters**

**CI**: confidence interval.

|  | **Intra-Class correlation** | **95% CI** | **Inter-Class correlation** | **95% CI** |
| --- | --- | --- | --- | --- |
| Left ventricle diameter | 0.93 | [0.89-0.99] | 0.93 | [0.70-0.91] |
| Right ventricle diameter | 0.87 | [0.65-0.98] | 0.91 | [0.64-0.98] |
| Aorta diameter | 0.96 | [0.70-0.98] | 0.90 | [0.65-0.96] |
| Main pulmonary artery diameter | 0.93 | [0.66-0.98] | 0.87 | [0.52-0.99] |
